# Quantitative phase microscopy of red blood cells during planar trapping and propulsion†
†Electronic supplementary information (ESI) available. See DOI: 10.1039/c8lc00356d


**DOI:** 10.1039/c8lc00356d

**Published:** 2018-08-22

**Authors:** Azeem Ahmad, Vishesh Dubey, Vijay Raj Singh, Jean-Claude Tinguely, Cristina Ionica Øie, Deanna L. Wolfson, Dalip Singh Mehta, Peter T. C. So, Balpreet Singh Ahluwalia

**Affiliations:** a Department of Physics and Technology , UiT The Arctic University of Norway , Tromsø N-9037 , Norway . Email: ahmadazeem870@gmail.com ; Email: balpreet.singh.ahluwalia@uit.no; b Department of Physics , Indian Institute of Technology Delhi , New Delhi 110016 , India; c Department of Mechanical & Biological Engineering , Massachusetts Institute of Technology , Cambridge , MA 02139 , USA; d BioSym IRG , Singapore-Alliance for Science & Technology Center , Singapore , Singapore

## Abstract

Red blood cells (RBCs) have the ability to undergo morphological deformations during microcirculation, such as changes in surface area, volume and sphericity. Optical waveguide trapping is suitable for trapping, propelling and deforming large cell populations along the length of the waveguide. Bright field microscopy employed with waveguide trapping does not provide quantitative information about structural changes. Here, we have combined quantitative phase microscopy and waveguide trapping techniques to study changes in RBC morphology during planar trapping and transportation. By using interference microscopy, time-lapsed interferometric images of trapped RBCs were recorded in real-time and subsequently utilized to reconstruct optical phase maps. Quantification of the phase differences before and after trapping enabled study of the mechanical effects during planar trapping. During planar trapping, a decrease in the maximum phase values, an increase in the surface area and a decrease in the volume and sphericity of RBCs were observed. QPM was used to analyze the phase values for two specific regions within RBCs: the annular rim and the central donut. The phase value of the annular rim decreases whereas it increases for the central donut during planar trapping. These changes correspond to a redistribution of cytosol inside the RBC during planar trapping and transportation.

## Abstract 2

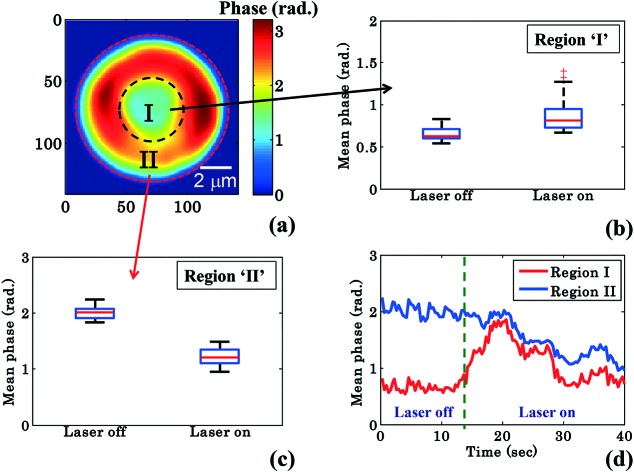
Here, we have combined quantitative phase microscopy and waveguide trapping techniques to study changes in RBC morphology during planar trapping and transportation.

## Introduction

Red blood cells (RBCs) are an essential component of blood and are responsible for oxygen delivery throughout the body.^1^ Human RBCs (erythrocytes) do not contain a nucleus and other sub-cellular organelles such as mitochondria, Golgi bodies and endoplasmic reticulum. Hemoglobin is the most abundant protein that is found within all RBCs and constitutes 95% of the RBC cytosolic proteins.^2^ Most of the RBC cytosol is water, which makes up about 70% of the total volume of a typical cell.^3^ Healthy RBCs have a biconcave shape with a mean diameter of ∼7–8 μm, with a thickness of ∼2.5 μm at the thickest point (annular rim) and 1 μm or less at the center (donut region). In order to exchange oxygen and carbon dioxide to and from the organs, RBCs must undergo significant deformation while passing through narrow micro-vessels such as those in the brain (diameter ∼2 μm) and through sieves in the spleen (diameter ∼1 μm).^4^–^6^ The biconcave shape and large surface area-to-volume ratio of RBCs facilitate their elastic flexibility,^7^ and the rearrangement of internal RBC scaffolding (cytoskeleton) enables RBCs to behave like a fluid and squeeze themselves through capillaries.^1^ A decrease or loss of RBC deformability is therefore associated with multiple diseases such as malaria, sickle cell anaemia, diabetes, cardiovascular disease, and hypertension. A loss of RBC deformability has also been reported during blood storage.^8^

Deformation of the red blood cells has been used for studying several diseases previously. Conventionally, the deformability of RBCs is studied using non-optical techniques such as micropipette aspiration and micro-fabricated channels.^9^–^12^ More recently, optical methods such as optical tweezers^13^–^16^ have been used to exert optical forces onto single RBCs and the response of optical forces/pressure on RBCs is studied using either bright field microscopy or fluorescence-based microscopy. Previous studies on RBC deformability have utilized techniques such as microfluidics^17^ or optical tweezers^15^,^18^ to simulate some of the forces encountered *in vivo*. With optical tweezers, a tightly focused laser beam uses force from the refractive bending of light to trap biological specimens in three-dimensions. Further, laser tweezers are more suited for studying single cells at a time.

**Fig. 1:**
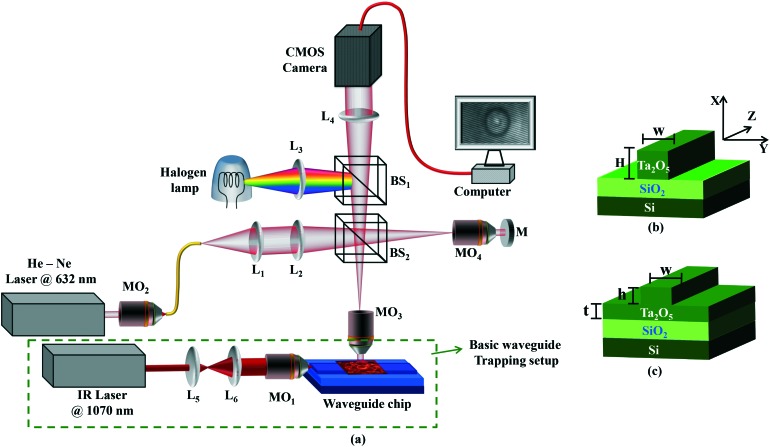
(a) Schematic diagram of the integrated waveguide trapping (green box) and the phase imaging setup. The sample is positioned on the top surface of the waveguide. L_1–6_: lenses, MO_1–4_: microscope objective lenses, BS_1–2_: beam splitters, and M: mirror. Fig. 1b and c show the schematic diagram of two optical waveguide geometries: (b) strip and (c) rib waveguides. Ta_2_O_5_: tantalum pentoxide, SiO_2_: silicon dioxide, Si: silicon substrate. The waveguide parameters are *w* = width, *H* = total thickness of the strip waveguide; *t* = slab region and *h* = rib region of the rib waveguide.

Over the past decade, planar waveguide trapping (WT) has emerged as an alternative tool to optical tweezers for on-chip manipulation and propulsion of micro-particles, gold nano-particles, and various biological objects (*e.g.* cells, bacteria and viruses) on the top of waveguide surfaces.^19^–^24^ In contrast to the focused beam of traditional optical tweezers, WT works using an evanescent light field, which is generated from totally-internally reflected (TIR) light guided through a path of high refractive index contrast on a semiconductor chip. Trapping occurs due to the exponential decay of the evanescent field relative to the waveguide surface, which generates a vertical gradient force (*F*_*x*_) that pulls a refractive objective (*e.g.* a cell) downwards towards the waveguide surface. A lateral gradient force (*F*_*y*_) is generated across the waveguide, acting as a restoring force to keep the cells trapped on the waveguide. The radiation pressure of light propagating through the waveguide results in a forward scattering force along the length of the waveguide (*F*_*z*_), and provides a forward push to propel the cells along the waveguide. Thus, while *F*_*x*_ and *F*_*y*_ stably trap the cells on top of the waveguide, *F*_*z*_ propels the cells slowly along the Z-axis shown in Fig. 1(b).

The capability of WT to trap large cell populations and its compatibility with microfluidics make it an ideal candidate to mimic the flow of RBCs in microcapillaries.^25^ Microfluidic devices have also been used for sorting and characterization of cells based on their size and stiffness contrast.^26^,^27^ Recently, it was shown that WT can be a useful tool for the assessment of the health or deformation of RBCs.^25^ In addition, this technique was also used to quantify the minute loss of RBC deformability during blood storage.^16^ More importantly, the optical forces imparted during waveguide trapping are in the order of 10 pN, in contrast to 50–400 pN (ref. 15, 25 and 28) optical forces being applied using laser tweezers. Besides being gentler, the optical forces imparted by the optical waveguide are spread over large surface areas in lateral dimensions (determine by the width of the waveguide) and are limited by the penetration depth of the evanescent field of the waveguide (typically 200 nm) in the axial direction. It was recently demonstrated that to detect the subtle loss of deformation of RBCs during blood storage lesion, it is necessary to impart minute optical forces.^25^ The optical force of around 10 pN imparted using the optical waveguide was found to be sufficient to detect a minute loss of deformability of RBCs within the first 10 days of blood storage.^25^

However, in the previously published paper, only bright field microscopy was used to investigate RBC deformation which did not provide any quantitative information during RBC deformation.^25^ The main motivation of this work is to integrate methodologies that can be used to a) trap and propel RBCs using minute optical forces from the planar waveguide; and b) can simultaneously obtain quantitative information about the RBCs during cell trapping and deformation.

Given the limitations of bright field microscopy, the combination of quantitative phase microscopy (QPM) and WT has high potential due to the ability to obtain label-free images and quantitative information about different parameters of biological cells. QPM is an emerging tool for studying weakly scattering and absorbing biological specimens.^29^ It provides information about the phase shift, *i.e.*, the optical path length (OPL) distribution produced by the specimen with respect to the surrounding medium. The optical path length contains the local information about the cell's refractive index and thickness. The phase shift is further used to determine various optical parameters of the specimen, such as cell's membrane fluctuations and non-aqueous content, *i.e.*, dry mass density of cells.^30^,^31^ Due to the ease of obtaining the interference pattern quickly, a number of non-common path and off-axis QPM methods based on coherent light sources have been developed for the quantitative phase measurement of living cells.^32^–^36^ In the off-axis set-up, the reference and the signal beams travel different light-paths. Furthermore, several other common-path QPM imaging techniques, such as diffraction phase microscopy (DPM),^37^ spatial light interference microscopy,^38^ and common-path diffraction optical tomography,^39^,^40^ have also been introduced for the quantitative measurement of different cell's parameters such as morphological, chemical, and mechanical parameters.

A phase microscopy set-up that is compatible for integration with the waveguide-trapping set-up was explored. In WT, the sample stage holding the waveguide is kept stationary during the image acquisition and the microscope is translated to capture the images of specimen during trapping and propulsion. However, in the conventional phase microscopy set-up, the microscope is kept stationary and only the sample is translated. Moreover, the phase microscopy set-up should be built in reflection mode as the waveguide chips are fabricated using opaque silicon substrates. In the present work, we developed a Linnik interferometer based reflection type QPM setup. As the Linnik interferometer is a compact reflection type QPM setup, its integration with WT is relatively straightforward. A Linnik interferometer has the capability to record off-axis holograms, which enables single shot Fourier transform based phase recovery of biological specimens and thus enables fast acquisition of the cell propulsion on top of the waveguide surface. By acquiring time-lapsed interferograms, the phase images and the morphological variation of the RBCs during the entire trapping process were obtained. The integrated WT and Linnik interferometric based QPM (WT-QPM) set-up was further employed for the quantitative measurement of various morphological parameters of the RBCs such as the surface area, volume, and sphericity during planar trapping and propulsion near the waveguide surface.

## Results and discussion

### Integrated waveguide trapping and quantitative phase microscopy (WT-QPM)

A QPM was combined with a WT system to simultaneously monitor and optically trap RBCs. The schematic diagram of the experimental set-up is shown in Fig. 1a. The green dotted box illustrates the conventional WT setup employed to trap and propel RBCs along the length of the waveguide. The remaining part of the experimental set-up depicts the off-axis quantitative phase microscope, which was used to monitor the changes in RBC's morphological parameters.

### Waveguide trapping

The green dotted box in Fig. 1a shows a 1070 nm laser which is first expanded using a pair of lenses (L_5_ and L_6_) to match the back aperture of an IR objective lens (MO_1_, 0.9 NA, 80×). The focused light is coupled into the waveguide, a process that is facilitated by mounting the objective on a three axis, piezo-controlled stage. The estimated power coupled into the waveguide for generation of the trapping field was 15 mW. Additional details about the waveguide trapping set-up can be found elsewhere.^24^,^25^,^41^

For efficient trapping, it is desirable to have a high intensity in the evanescent field. This is achieved by fabricating thin optical waveguides (150 nm thick) made of high refractive index contrast materials. Here, optical waveguides were made of tantalum pentoxide (Ta_2_O_5_),^42^ which has a refractive index of 2.1. The Ta_2_O_5_ platform possesses low auto-fluorescence and low propagation losses and has been previously employed for trapping of living cells.^25^

For on-chip manipulation, generally two different waveguide designs are used: strip and rib waveguides (Fig. 1(b and c)). Strip and rib waveguides were fabricated by sputtering a guiding layer Ta_2_O_5_ onto a silica (Si) layer followed by photolithography and argon ion-beam milling.^42^ For strip waveguides, the Ta_2_O_5_ layer (*n*_core_ = 2.1) had a thickness ‘*H*’ of 220 nm, and was completely etched down to the Si layer (*n* = 1.45). For the rib waveguides, the Ta_2_O_5_ layer is only partially etched down with different thicknesses ‘*h*’ of 4, 8, 20, 50, and 150 nm leaving a final slab thickness of ‘*t*’. A similar procedure was followed for the fabrication of 20 and 220 nm height waveguides having different widths ‘*w*’ of 2.5, 3, 4, 5, and 10 μm. More details on the optimization of waveguide fabrication can be found elsewhere.^43^

### Quantitative phase microscopy

The upright QPM based on the principle of a Linnik-interferometric microscope, captured the real-time behavior (*i.e.*, attraction and propulsion) of the trapped RBCs on the waveguide. A He–Ne laser (power ∼5 mW, *λ* ∼ 632 nm) was used to acquire good contrast interference patterns. The light beam coming out of the laser was coupled into the single mode fiber using the 20× objective lens MO_2_. The fiber carried light at the input port of the microscope as shown in Fig. 1(a). The output port of the fiber worked as a pinhole, employing a diverging beam, which further collimated using lens L_1_. The collimated laser light beam was focused at the back focal plane of objective lens MO_3_ (0.7 NA, 60×) in the sample arm, which sends a collimated beam at the output port of the objective lens. The sample is illuminated by a collimated beam, which avoids the serious phase error generated due to the converging or diverging nature of the light beam. Beam splitter BS_2_ splits the input beam into two and sends one of the beams towards the reference mirror, whereas the other one towards the sample. After getting reflected from the sample and the mirror, both the beams are combined at the same beam splitter and projected an off-axis hologram at the camera plane. Finally, a spatially modulated hologram of the sample was captured using a high speed CMOS (Hamamatsu ORCA-Flash4.0 LT, C11440-42U) image sensor. The image acquisition rate was set to 30 frames per second.

To measure the capability of the proposed technique for the quantification of small morphological changes, experiments were conducted on a standard flat mirror and a waveguide substrate. The spatial phase/height measurement sensitivity of the QPM setup was characterized by acquiring interferometric images using a flat mirror and a waveguide substrate as a test specimen. The peak to valley (P-V) spatial phase^29^ of the system for the standard flat mirror is found to be equal to 143 mrad, whereas it is measured to be equal to ∼313 mrad during optical trapping, which corresponds to the height measurement accuracy of 16–20 nm. This is further confirmed experimentally by employing the system for the height measurement of the 20 nm rib height waveguide. The 20 nm rib height of the optical waveguide is not visible, *i.e.*, embedded with phase noise of the system, in the reconstructed RBC phase map (see ESI† Fig. S1–S3). A systematic study about the measurement capability, *i.e.*, *z* resolution, of the system can be found in the ESI† note. Fourier transform-based image processing was used to retrieve the phase information of the specimen from the interferometric images. Additional information about retrieval of phase information is discussed in the ESI.†

### Reduction of waveguide induced phase errors

In conventional laser tweezers, the RBCs are trapped in three-dimensions and typically away from the substrate; thus getting the QPM is relatively straightforward.^44^ Conventional optical tweezers utilize a tightly focused laser beam for cell manipulation which may damage the biological specimens. However, in waveguide trapping, the RBCs are trapped directly on top of the waveguide surface adding the phase value of the waveguide core into the RBC phase. The phase microscope provides collective information of both the RBCs and waveguide phase. Therefore, in order to obtain noise-free phase information of RBCs, the waveguide phase information has to be subtracted from the total phase value. Subtraction of the unwanted waveguide phase information would require extra post-processing, making the method cumbersome and time consuming. The subtraction of the waveguide phase was tried but was found to be challenging as RBCs are continuously propelled along the length of the waveguide, thus constantly changing the background information (waveguide phase). Although the fabrication process of the waveguide has been optimized,^42^ the high sensitivity of our phase microscopy (16–20 nm) can easily pick up slight non-uniformity in the waveguide core thickness and randomly varying sidewall roughness at the waveguide edges. The problem increases further as the waveguide core is made of a high refractive index material (Ta_2_O_5_) that generates high index contrast (Δ*n*) with the buffer medium (Δ*n* = 0.79).

To overcome this challenge, we have systematically investigated different waveguide geometries (rib and strip) and parameters (width and thickness). Fig. 1(b and c) show the schematic diagram of strip and rib waveguides. The core layer is completely etched for a strip waveguide while it is only partially etched for a rib waveguide as shown in Fig. 1c. A rib waveguide has a slab region (depicted as *h* in Fig. 1c) beneath the shallow rib region (depicted as *t* in Fig. 1c). The total thickness of a strip and a rib waveguide is denoted by *H* and *t* + *h* in Fig. 1(b and c), respectively. Both strip and rib waveguides have been used in the past for optical trapping applications.^22^,^24^ ESI† Fig. S4 and S5 show the phase map of RBCs placed on top of rib and strip waveguides with varying widths. Shallow rib waveguides are more suitable than the strip waveguides due to reduced phase noise of the waveguide core layer. To systematically study the influence of the waveguide core on phase noise, experiments on waveguides with varying waveguide rib height (*h* in Fig. 1c) were performed. ESI† Fig. S6 shows the phase map of RBCs placed on top of a rib waveguide as a function of the rib height for a given width of 2.5 μm. The waveguide with a rib height (*h*) of up to 50 nm could be used to reconstruct the RBC phase without appreciable phase noise from the waveguide. The intensity in the evanescent field decreases with decreasing rib height. Thus, a compromise must be made such that there is a high intensity in the evanescent field with little phase noise from the waveguide core. The shallow rib waveguide avoids unwanted phase information and consequently the phase artifacts during cell propulsion. In addition, the shallow rib height was shown to have lower propagation losses as compared to the strip waveguides.^42^ The rib waveguide with a rib height of 20 nm and a width of 3 μm was used for the remaining work.

### Defocus correction for accurate assessment of phase maps

To obtain the morphological parameters of the trapped RBCs, precise retrieval of the phase map of the RBCs is recommended. The phase value of the RBCs will alter if there is any defocusing issue during the dynamic trapping phenomena. In addition, a relatively high magnification and high numerical aperture (60×, 0.7 N.A.) objective lens with a small depth of field was used to acquire images. A small defocusing during the data acquisition of the trapping phenomena will result in phase error. There can be several possibilities of RBC defocusing during trapping/propulsion: (1) a slight angle in the waveguide platform, (2) a tilt in the whole upright interference microscope, and (3) a downward pull of the RBCs towards the waveguide surface when the trapping laser is switched on. Therefore, it is required to numerically refocus the object field information for accurate phase measurement of RBCs. A previously proposed focus correction algorithm based on the angular spectrum propagation^31^ approach was employed in post-processing for an accurate assessment of RBC phase maps (see ESI† Fig. S7), thus removing the defocusing issue.

In order to determine the defocus distance, first, the complex field retrieved from the Fourier transform method is numerically propagated across the range of –15 μm to +15 μm. Then amplitude variance is calculated corresponding to each propagated field and plotted as a function of the propagation distance. The minimum of the amplitude variance *vs.* propagation distance plot gives information about the defocus distance or true focal plane of the objective lens. Further, the complex field is propagated by the above calculated defocus distance using the angular spectrum approach to acquire accurate RBC phase information. Similar steps were followed for each interferometric frame of the recorded movie. The detailed information about the algorithm can be found elsewhere.^45^ Further, the influence of defocus on the quantitative phase measurement of RBCs is shown in ESI† Fig. S7.

### Quantification of the RBC phase value during planar trapping

**Fig. 2:**
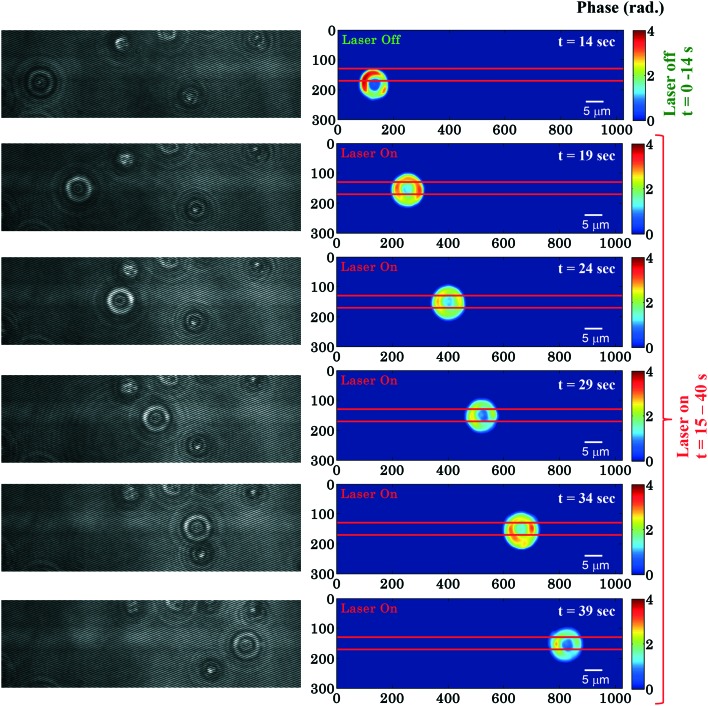
Time lapsed interferometric and quantitative phase images of RBCs propelling along the length of the waveguide. The horizontal red color lines are drawn to depict the position of the waveguide. The color bar is in radians. Associated ESI† Movie (Video S2). The phase of the trapped RBCs is only computed in the right column.

Fig. 2 shows time-lapsed interferometry and phase images of RBCs during planar trapping. The trapping laser was off for the first 14 s and it was switched on between 15–40 s. When the laser beam is switched on, the RBCs on top or in the near vicinity of the waveguide are trapped and propelled along the length of the waveguide. The interferometric movie (see ESI† Video S1) was acquired in order to measure real-time phase changes during the entire trapping phenomena. The time-lapsed phase images of the RBCs (see ESI† Video S2) were obtained from the recorded movie using the Fourier transform based phase recovery algorithm. Additional information about the extraction of the phase is provided in the ESI† note. The phase variation of RBCs during planar trapping can be seen from Fig. 2.

**Fig. 3:**
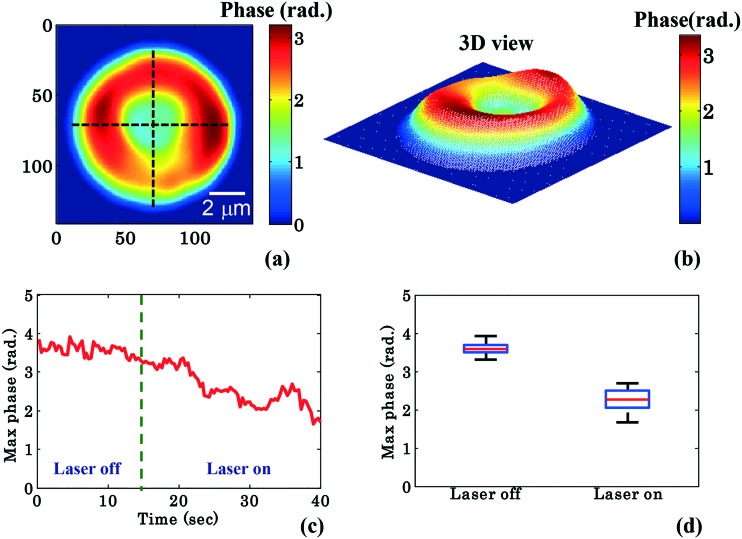
Quantitative phase imaging highlights the change in RBC phase value during waveguide trapping and propulsion. (a) shows the 2D view of the RBC phase image being divided into four quadrants using two crossed black lines, (b) corresponding 3D view, (c) variation of the maximum phase values as a function of time, (d) whisker box plot of the maximum phase values (*φ*_max_) for laser off (0–14 s frames) and on (26–40 s frames) conditions for a single movie (experiment). The central red lines indicate the median, and the bottom and top sides of the blue box indicate the 25th and 75th percentiles, respectively. The black lines extended vertically from the blue boxes specify extreme data points without outliers.

To further quantify the phase variation and to minimize the experimental and processing errors, for example, due to hot spots in the phase images, the RBC was divided into four quadrants as shown in Fig. 3a. The maximum phase (*φ*_max_) value of each quadrant is calculated and then the average maximum phase value of the all four quadrants is determined, thus reducing the errors from any hot-spots. Fig. 3b depicts the 3D view of the reconstructed RBC phase map.

Fig. 3c shows the variation of the *φ*_max_ value of the RBC as a function of time of the experiment shown in Fig. 2. During the first 14 s when the laser was switched off, only a small variation in the *φ*_max_ value of the RBC (∼0.14 rad) was observed. This small phase variation could be due to Brownian motion or thermal fluctuation of the RBC. At *t* = 14 s, the laser is switched on and the *φ*_max_ value of the RBC starts to decrease gradually. At the end of the movie, *i.e.* at *t* = 40 s, the maximum phase value of the RBC decreased to ∼1.92 rad (Fig. 3c). The difference (*φ*diffmax = *φ*trappedmax – *φ*untrappedmax) between the *φ*_max_ value between the trapped (*φ*trappedmax) and untrapped *φ*untrappedmax RBCs is found to be ∼1.35 rad. It is quite evident that the decrease in the *φ*_max_ value (∼1.35 rad) due to the trapping laser is much larger compared to the phase variation due to the Brownian motion or thermal fluctuation of the RBC (∼0.14 rad) when the laser was switched off.

Fig. 3d shows the whisker box plot of the maximum phase value (*φ*_max_) for laser off and on conditions of the same cell. The whisker box plot highlights that when the laser is switched on, the maximum phase value of the trapped RBC is decreased. The maximum phase of the RBC is found to be 3.59 rad and 2.24 rad when the laser is switched off and switched on, respectively, as illustrated in Fig. 3d. The recovered maximum phase values of the trapped and untrapped RBCs at different time intervals are also exhibited using a bar plot in ESI† Fig. S9a.

RBCs are bi-concave in shape; the maximum height of the RBC rim is around 2 μm and that of the central donut is around 0.5 μm. Therefore, the maximum phase value of the RBC is at the rim of the RBC. Assuming that the refractive index of the RBC does not change during waveguide trapping, the decrease of the maximum phase value indicates that the RBC is pushed downwards during the planar trapping and propulsion. This is also in accordance with previously published data, where three-dimensional finite element simulation showed that the intensity gradient generated by the exponentially decaying evanescent field of the waveguide attracts the biconcave shaped RBC towards the surface and presses it downwards. A previous study has explained the phenomenon of RBC deformation on top of the waveguide surface using the finite element numerical simulations of the optical forces and the optical pressure of trapped RBCs.^25^ It was reported that the net effect of the force density (*i.e.* pressure multiplied with surface normal) was to press the cell downwards and straighten (flatten) the part of the cell that is overlapping with the evanescent field and make it parallel to the waveguide. When this part of the cell becomes straight, the rest of the cell deforms and a change in the shape occurs. The elastic membrane of the RBC plays a crucial role in spreading the localized optical pressure over the entire cell. Further details can be found in the ESI.† However, in previous work^23^ only bright field microscopy was used and therefore no quantitative information was obtained. The present method maps the pseudo 3-D phase value of the RBC during planar trapping and could further elucidate the process of RBC deformation during planar trapping. Taken together with the previously published work,^25^ it can be suggested that the main effect of the waveguide trapping force is to pull the cell downwards and thus decrease the maximum phase.

**Fig. 4:**
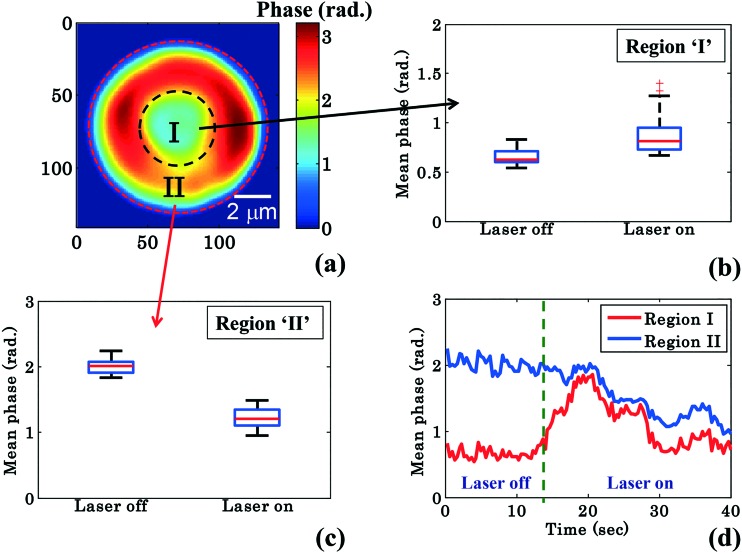
Quantitative phase imaging highlights the change in the RBC phase value during waveguide trapping and propulsion. (a) Shows the RBC phase image divided into region I (inside black dotted circle), *i.e.*, donut and region II (annular region between red and black dotted circle), *i.e.*, non-donut region. Whisker box plot (b and c) of the mean phase values of the time lapsed frames (shown in Fig. 2) of region I and region II for a single movie (experiment). The central red lines indicate the median, and the bottom and top sides of the blue box indicate the 25th and 75th percentiles, respectively. The black lines extended vertically from the blue boxes specify extreme data points without outliers, and ‘+’ symbols in red color are plotted for outliers. (d) Variation of the mean phase values of region I and region II as a function of time.

RBCs are easily deformable; therefore the cytosol within the RBC could re-distribute between the donut and the annular rim during planar trapping. To shed more light on this process, the RBC phase images were divided into two different regions: region I: the central donut region (inside black circle) and region II: the annular rim of the RBC (region between outer red and inner black circle) as depicted in Fig. 4a. The edge detection image processing technique is employed for finding the boundaries of the RBC phase images by setting a threshold value (∼30% of the maximum value) using MATLAB. Edge detection is used for RBC phase image segmentation into three different regions: (1) region I, (2) region II, and (3) outermost region (Fig. 4a). In order to observe the change in the mean phase value of the RBC during the transportation, the shape as well as the area of segmented region ‘II’ was kept constant while analyzing the reconstructed phase maps of one experimental series. Finally, the mean phase values of regions ‘I’ and ‘II’ were measured for both laser off and on conditions.

Fig. 4b and c show the mean phase value box plots of the trapped and untrapped RBCs (shown in Fig. 2) for region I and II, respectively. To understand the influence of trapping forces on RBCs, the recovered time-lapsed mean phase values of the trapped and untrapped RBCs for both regions are also presented by bar plots as illustrated in ESI† Fig. S9b and c. For the measurement of the above values, 40 frames each (from Movie S1†) were selected from the single interferometric movie when the laser was switched on and off. The chosen time points for laser off was 0–14 s and for laser on 26–40 s with an interval of 0.33 s each. The image acquisition was set to 30 frames per second. Interestingly, the mean phase value of region ‘I’ is increased by ∼32%, whereas the mean phase value of region ‘II’ is decreased by ∼40% during RBC trapping. For region ‘I’, the average mean phase obtained from the 40 frames used was 0.65 ± 0.07 rad and 0.86 ± 0.18 rad for trapping laser off and on conditions, respectively. Meanwhile, for region ‘II’ the average mean phase obtained from the 40 frames used was 2.00 ± 0.10 rad and 1.21 ± 0.14 rad for trapping laser off and on conditions, respectively.

The difference (*φ*diffmean = *φ*trappedmean – *φ*untrappedmean) between the mean phase values of the time lapsed frames of the trapped (*φ*trappedmean) and untrapped (*φ*untrappedmean) RBCs for both regions was found to be 0.21 rad for region I and –0.79 rad for region II, respectively. The positive and negative values of *φ*diffmean for region ‘I’ and region ‘II’ suggest the re-distribution of the cytosol inside the RBC (which mostly consists of hemoglobin) from region ‘II’ (annular ring) to region ‘I’ (donut) during planar trapping. The variation of mean phase values of region I and II as a function of time is illustrated in Fig. 4d.

**Fig. 5:**
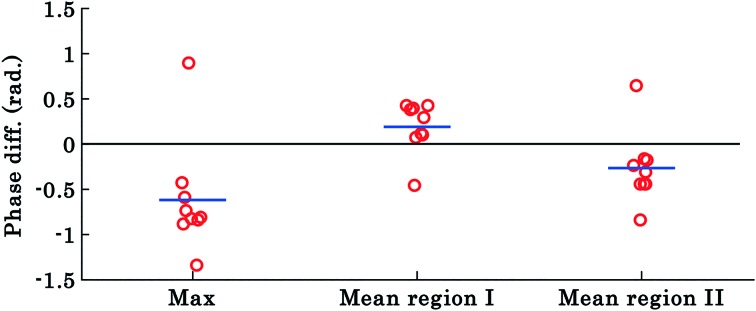
The scattering plot of the difference between maximum phase values and mean phase values of region ‘I’ and ‘II’ of trapped and untrapped RBCs corresponding to nine different experiment series (movies). Each circle represents the difference between phase values from a RBC from different experiment series and the horizontal blue line depicts the mean of the nine data points. The results highlight that the maximum and mean phase values of region II of the RBC decrease, whereas the mean phase value of region I increases during waveguide trapping.

The results shown in Fig. 3 and 4 correspond to a single experiment and the results from additional experiments (9 in total) are presented in Fig. 5. The phase difference is calculated by taking the difference between phase values corresponding to the trapped and untrapped RBCs, *i.e.*, using the following expression: *φ*diffmax or mean = *φ*trappedmax or mean – *φ*untrappedmax or mean. The overall trends of the maximum and mean RBC phase values (region I and II) during waveguide trapping (see Fig. 5) were similar to the results obtained for a single movie presented in Fig. 3 and 4. Similar to the results obtained in Fig. 3c, the maximum phase value of the RBCs also decreases during waveguide trapping which was observed for other experiments (see Fig. 5).

The difference *φ*diffmean plot of the RBC phase values corresponding to region I and II before and after trapping from the additional 9 experiments is presented by the scattering plot in Fig. 5. The *φ*diffmean is calculated from 40 different frames under laser on and off conditions at an interval of 0.3 s. The red circles in the scattering plot correspond to *φ*diffmean for different experimental movies. The horizontal blue line corresponds to the average value of nine different movies for both laser off and on conditions.

### Measurement of the elongation index

**Fig. 6:**
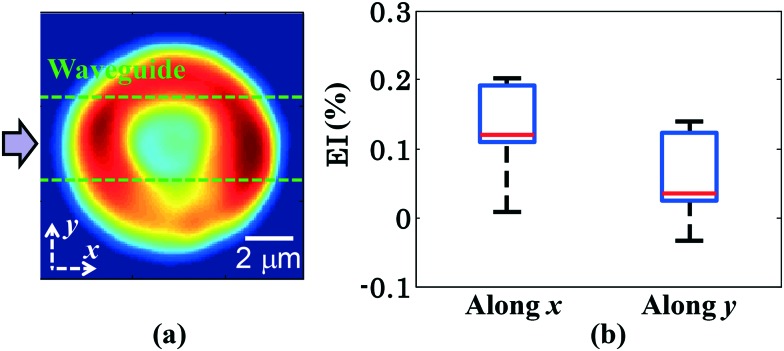
The box plot of the EI of human RBCs during waveguide trapping for 9 different experimental series. (a) 2D view of the RBC's recovered phase map. (b) Whisker box plot of the RBC's EI along the *x* and *y* axes during waveguide trapping. The axes are defined in Fig. 6a.

The measurement of the elongation index (EI) is crucial for the comparison of the deformability of distinct cell lines or, the same cell line at various diseased states. The EI of RBCs along the *x* and *y* axes during optical waveguide trapping is quantified using the following expression:
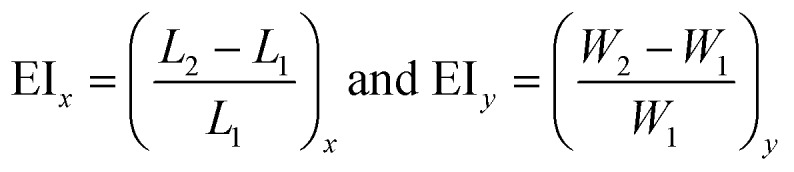
where *L*_1_, *W*_1_ and *L*_2_, *W*_2_ are the lengths and widths of the untrapped and trapped RBCs, respectively. Fig. 6a represents the reconstructed phase image of a human RBC with two green dotted horizontal lines depicting the position of the waveguide. The whisker box plot of the RBC's EI along the *x* and *y* directions for 9 different experimental series is illustrated in Fig. 6b. The bar plot of the RBC's EI along the *x* and *y* directions for all 9 experiments is exhibited in ESI† Fig. S10. For the calculation of the EI along the *x* and *y* directions, the RBC's phase map is projected onto the *x*–*y* plane. Then the horizontal (*L*_1_ or *L*_2_) and the vertical (*W*_1_ or *W*_2_) extents of the projected area of the untrapped and trapped RBCs' phase maps are calculated along the both *x* and *y* directions and further utilized for the measurement of the EI_*x*_ and EI_*y*_. The mean values of the EI_*x*_ and EI_*y*_ are found to be equal to 0.1226 and 0.0578 along the *x* and *y* directions, respectively. It can be seen that the degree of RBC elongation in the trapped state is found to be high along the *x*-axis, *i.e.*, along the length of the waveguide (direction of the RBC's propulsion), as compared to the *y*-axis, *i.e.*, along the width of the waveguide.

### Characterization of the RBC's morphological changes

**Fig. 7:**
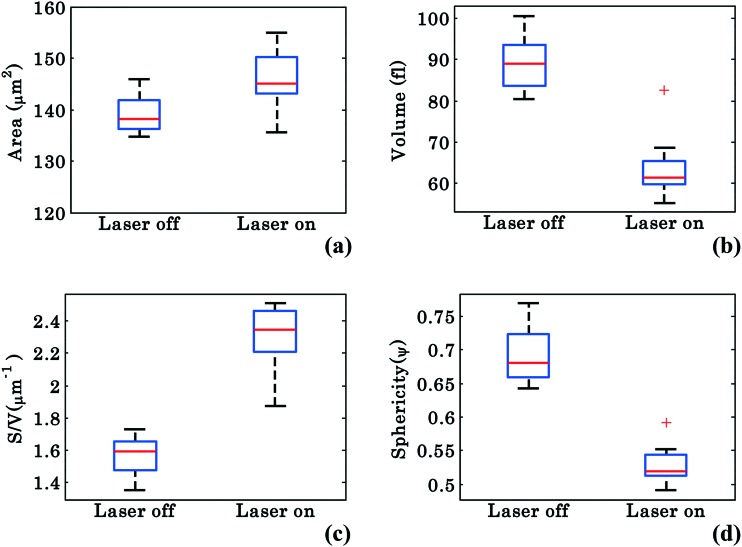
Morphological parameters of RBCs for a single interferometric movie. Whisker box plots of RBCs: (a–d) surface area, volume, surface area-to-volume ratio (*S*/*V*), and sphericity under trapped and untrapped conditions.

In order to better characterize the morphological changes occurring in the cell, phase maps were converted into spatial maps and the cells' morphology was quantified according to the calculations described in Materials and methods. The parameters analyzed include the surface area (*S*), volume (*V*), surface-to-volume ratio (*S*/*V*), and sphericity (*ψ*), which is defined as the ratio of the volume of a perfect sphere to the actual volume of the cell with the same surface area. Similar to Fig. 3, these values were chosen from 40 different frames (from Movie S1†) at an interval of 0.3 s starting at 0–14 s for laser off and starting at 26–40 s for laser on conditions. Fig. 7(a–d) show the whisker box plots for these values during waveguide trapping of RBCs as shown in Fig. 2. The results show an increase in both *S* and *S*/*V* when the laser is switched on, but a decrease in both *V* and *ψ*. ESI† Fig. S8 shows further evidence for this volume change for a trapped RBC.

The decrease of *V* and *ψ* combined with the increase of *S* indicates that the shape of the RBC approaches a plano-concave shape during planar trapping. Under the assumption that the refractive index of the RBCs remains constant during the waveguide trapping, the decrease in the RBC internal volume when the trapping laser is switched on could possibly be occurring due to the elongation of the RBC under the stress condition, *i.e.*, out flux of cell water and solute.^46^ It was previously reported that stressing the RBC membrane can lead to the opening of pores that allow exit of intracellular materials.^47^ At the same time, the *S*/*V* of the cell increases (Fig. 7c), indicating that the shape of the RBC is elongated during planar trapping (see Fig. 6). The mean values with standard deviation (SD) of the surface area, volume, surface area-to-volume ratio, and sphericity of RBCs under trapped and untrapped conditions can be found in Table 1. During waveguide trapping, the surface area increased by 5%, the volume decreased by 28%, the *S*/*V* ratio increased by 44% and the sphericity decreased by 23%. These values are calculated only for a single experimental movie.

**Table 1:** Morphological parameters of the RBC *i.e.* surface area, volume, *S*/*V*, and sphericity during trapped and untrapped conditions, corresponding to the experiment shown in Fig. 2. The description of the quantification methods is discussed in the Materials and methods section

No.	Morphological parameters	Laser off	Laser on
1.	Surface area ‘*S*’ (μm^2^)	138.96 ± 3.64	146.00 ± 5.70
2.	Volume ‘*V*’ (fl)	89.04 ± 6.51	63.93 ± 7.93
3.	*S*/*V* (μm^–1^)	1.56 ± 0.12	2.30 ± 0.19
4.	Sphericity ‘*ψ*’	0.69 ± 0.04	0.52 ± 0.03

**Fig. 8:**
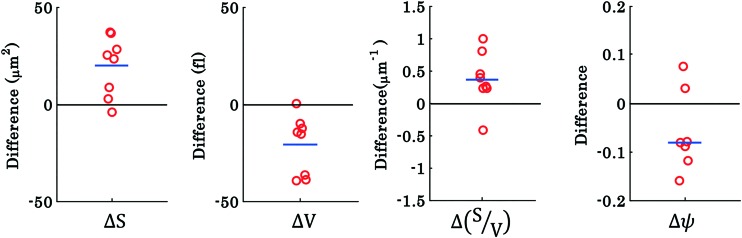
Scattering plots of the difference between morphological parameters of RBCs for nine different interferometric movies: (a–d) surface area, volume, surface area-to-volume ratio (*S*/*V*), and sphericity, for laser off (untrapped) and laser on (trapped) conditions. Each circle represents the value from new cells from different experiment series and the horizontal blue line depicts the mean of the nine data points.

To investigate the morphological alterations statistically in the RBCs while propelling them on the top of the waveguide, we quantitatively analyzed the phase maps and subsequently retrieved important cell's morphological parameters such as the surface area, volume, *S*–*V* ratio, and sphericity, for nine different experiments. Fig. 8 shows the scattering plots of the difference between morphological parameters such as the surface area, volume, *S*–*V* ratio, and sphericity, for the trapped and untrapped RBCs for 9 different experiment series. These values were chosen from 40 different frames at an interval of 0.3 s when the laser is off and on for the individual experiments. Each red circle shown in Fig. 8 corresponds to an individual experiment. The morphological parameter (MP) difference is calculated by taking the difference between their values corresponding to trapped and to untrapped conditions, *i.e.*, using the following expression: 
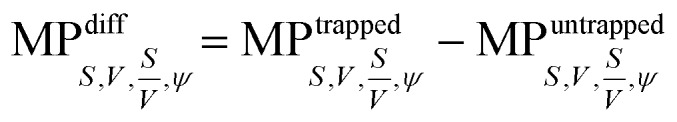
. The positive and negative values indicate either an increase or a decrease in the parameter's value respectively during trapping. The values of the different morphological parameters corresponding to different experiment series followed a similar trend to that presented in Fig. 7, *i.e.* during waveguide trapping, the surface area and the volume of the RBC is slightly increased and decreased, respectively. This leads to an increase in the surface-to-volume ratio and a decrease of RBC sphericity with waveguide trapping.

## Conclusion

In this work, we developed a methodology to trap and propel RBCs using planar optical waveguides and used quantitative phase microscopy to simultaneously detect the response of RBCs during planar trapping and propulsion. The main motivation was to develop a methodology for quantitatively studying the sub-cellular re-orientation of RBCs when exposed to minute optical forces from the waveguide surface, typically in the range of 10 pN.^23^ The incorporation of QPM with waveguide trapping (WT) allows obtaining quantitative information of biological samples under stress from the optical forces. Several challenges associated with successful implementation of WT with QPM have been addressed, such as reducing the unwanted phase from the waveguide (see ESI† Fig. S4–S6) and solving the defocusing issue that can lead to inaccurate phase measurement. It is important to highlight that the focus correction algorithm plays a vital role in the precise measurement of morphological parameters (such as the surface area, volume and sphericity) of dynamic samples, like RBCs during planar propulsion. After utilizing the focus correction algorithm, the experimental data was analyzed to quantify the maximum and the mean phase values of the trapped and untrapped RBCs. The retrieved phase map of the RBC before and after trapping is used as an indicator of the redistribution of cytosol inside the RBCs. It was found that the mean phase value for the central region of the RBCs increased and the phase value for the annular rim regions of the RBCs decreased. The results suggest that the cytosol content redistributed from the annular rim region (region II) towards the central donut region (region ‘I’) during planar trapping. The RBCs are pulled downwards towards the waveguide surface and the mean phase value becomes smaller during the surface transportation (see ESI† Fig. S8). In addition, the changes in the morphological parameters such as the surface area, volume, and sphericity were obtained to further understand the effect of the optical forces generated by the waveguide on the cells. Increased surface area and *S*/*V* and decreased volume and sphericity of the trapped RBC as compared to the untrapped RBC were obtained using the proposed methodology.

Although our motivation to integrate waveguide trapping with quantitative phase microscopy was driven by RBC application, the proposed methodology can also be employed for studying cellular changes in other cells (such as bacteria and platelets) when exposed to optical forces. The stiffness of the biological cells can further lead to analysis of several diseases like sickle cell anemia, malaria, *etc.* The proposed approach can be utilized for the quantitative assessment of the health of the RBC, for example in blood storage lesion.^23^

The spatio-temporal phase sensitivity of the present microscope is around 40 and 20 mrad, which can be increased further by employing a common path interferometer and a low spatial coherent monochromatic light source. The spatio-temporal phase sensitivity of the phase microscope has been improved by using a compact Mirau interferometric objective lens and a synthesized pseudo thermal light source previously.^34^,^48^ This can further lead to an increase in the phase measurement accuracy of biological objects during planar trapping and propulsion.

The integrated waveguide chip platform is compatible with other integrated on-chip optical functions such as micro-fluidics and on-chip nanoscopy,^49^ sensing^50^ and spectroscopy.^51^ Recently, chip-based nanoscopy^34^ has been demonstrated by employing a similar waveguide chip to that used in this work. Therefore, our existing WT-QPM set-up can be easily combined with on-chip fluorescence nanoscopy techniques for simultaneously acquiring super-resolved fluorescence images and the phase image of the biological specimen.

## Materials and methods

### Human subjects

Three healthy volunteers (all men, co-authors of this study) were recruited for minimally invasive venous puncture for whole-blood specimens. The subjects were fully aware of the experiments and have signed a written informed consent. The purpose of this project is outside the statutory area of the Health Research Act, and did not require an ethical approval according to the Regional Ethics Committee (REK).

### Blood sample preparation

Fresh blood samples were collected in EDTA (ethylene diamine tetra acetic acid) coated tubes to prevent blood coagulation. The blood sample was immediately mixed with phosphate buffered saline solution (PBS) and centrifuged 3× at 600*g* for 10 minutes to isolate RBCs from the other blood components (white blood cells and plasma). Isolated RBCs were washed 2× with isotonic sucrose solution (0.25 M) to prevent the RBCs from sticking onto the waveguide surface. The RBC samples were stored at 4 °C prior to measurements. The RBCs were diluted in isotonic sugar solution at a concentration of 0.1% RBC, and a small volume of RBC solution was pipetted into a biocompatible 3 × 3 mm^2^ polydimethylsiloxane (PDMS) chamber, which was sealed from the top with a 170 μm thick glass cover slip, to avoid leaking and evaporation of the medium during the experiment.

### Characterization of the RBC's morphological parameters

The height map *h*(*x*, *y*) of the RBC is obtained from its phase map *φ*(*x*, *y*) using the formula *h*(*x*, *y*) = 
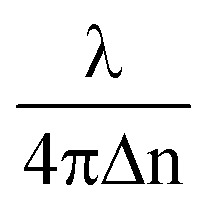
 *φ*(*x*, *y*), for the calculation of the surface area and volume of cells (where *λ* = 632 nm is the wavelength of illumination and Δ*n* = *n*_c_ – *n*_m_, the difference between the refractive index value of the cell ‘*n*_c_’ and the surrounding media ‘*n*_m_’). The refractive index values of *n*_c_ and *n*_m_ are assumed to be equal to 1.39 (ref. 52) and 1.34 (the refractive index of isotonic sucrose media), respectively, for calculating the cell's height map. Once the height of the cell was obtained, the volume of the cell was calculated by integrating the height map into the projected area as *V* = ∫∫ *h*(*x*, *y*)d*x*d*y* for both laser off and on conditions.

Further, the surface area of the RBC was measured using Monge parametrization defined as 
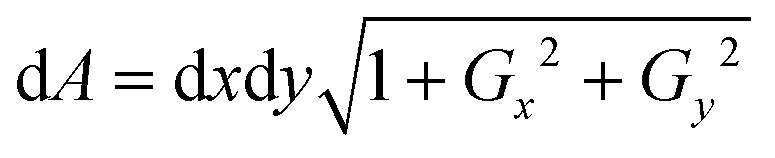
, where d*A* is the area element of the RBC surface, *G*_*x*_ and *G*_*y*_ are the gradients along the *x* and *y* directions, and d*x* and d*y* are the calibrated pixel width along the *x* and *y* directions, respectively.^29^ The surface area ‘*S*’ of the RBC is then the sum of all the area elements and projected area (see the ESI† note and Fig. S11). Next, the sphericity ‘*ψ*’ of trapped and non-trapped RBCs was determined, whose values lie between 0 (for a laminar disk) and 1 for a perfect sphere. It is defined as the ratio between the surface area (*S*) of a sphere with the same volume as the cell, to the actual surface area of the cell^29^,^53^ and is calculated as *ψ* = 4.84 v^2/3^ s^–1^.

## Author contributions statements

B. S. A. conceived the project and B. S. A., D. S. M., V. R. S. and P. S. supervised the research. A. A. and J. C. T. built the set-up. A. A. performed the majority of the experiments and V. D. and J. C. T. assisted during the experiments. C. I. Ø., and D. L. W. isolated and prepared the cells. A. A. generated the images, analyzed the data, and prepared the figures. A. A., D. L. W., and B. S. A. wrote the manuscript. All the authors reviewed the manuscript.

## Conflicts of interest

All authors declare no competing financial interests.

## Supplementary Material

Supplementary information

Supplementary movie

Supplementary movie
