# Supplementary material for: Quantitative phase microscopy of red blood cells during planar trapping and propulsion
Source: Lab Chip. 2018 Aug 22;18(19):3025–36. doi: 10.1039/c8lc00356d (PMC6161620; doi:10.1039/c8lc00356d)
Supplement: Supplementary file 1 [file LC-018-C8LC00356D-s001.pdf]

Electronic Supplementary Material (ESI) for Lab on a Chip.
This journal is © The Royal Society of Chemistry 2018

Quantitative phase microscopy of red blood cells during planar trapping and propulsion

Azeem Ahmad[1, 2,a], Vishesh Dubey[1, 2], Vijay Raj Singh[3], Jean-ClaudeTinguely[1], Cristina Ionica Øie[1], Deanna L. Wolfson[1],Dalip Singh Mehta[2], Peter T. C. So[3,4] and BalpreetSingh Ahluwalia[1*]

Supplementary Information for Ahmad et al., "Quantitative phase microscopy of red blood cells during planar trapping and propulsion"

Video Caption:

Supplementary video S1 –Time- lapsed interferometric imaging of human RBC propelling along the length of waveguide.

Supplementary video S2–Time-lapsed recovered phase movie using interferometric movie shown in the supplementary video S1.

Supplementary Note

Phase retrieval algorithm:

The Fourier transform based phase retrieval algorithm was used to study the structural changes in RBC while propelling it onto the surface of the waveguide. The 2D intensity variation of the image hologram can be expressed as follows[1, 2]:

$$f(x,y) = a(x,y) + b(x,y)\cos\left[2\pi i(f_x x + f_y y) + \phi(x,y)\right] \qquad (1)$$

where $a(x,y)$ and $b(x,y)$ are the background (DC) and the modulation terms, respectively. Spatially-varying phase $\phi(x,y)$ contains information about the specimen. f_x, f_y are the spatial carrier frequencies of interferogram along x,y axes. In practical applications, it is envisaged that $a(x,y)$, $b(x,y)$ and $\phi(x,y)$ are slowly varying functions compared to the variation introduced by the spatial carrier frequencies f_x, and f_y.

The above intensity modulation can be rewritten in the following form for convenience

$$f(x,y) = a(x,y) + c(x,y)\exp\left[2\pi i(f_x x + f_y y)\right] + c^*(x,y)\exp\left[-2\pi i(f_x x + f_y y)\right] \qquad (2)$$

where

$$c(x,y) = b(x,y)\exp\left(i\phi(x,y)\right) \qquad (3)$$

The Fourier transform of Eq. 2 is given as follows:

$$F(\xi_x,\xi_y) = A(\xi_x,\xi_y) + C(\xi_x - f_x,\xi_y - f_y) + C^*(\xi_x + f_x,\xi_y + f_y) \qquad (4)$$

The term $A(\xi_x,\xi_y)$ is simply a background (DC) term at the origin in the Fourier plane. The term $C(\xi_x - f_x,\xi_y - f_y)$ corresponds to +1 order term contains information about the object and situated at $(+f_x, +f_y)$. Similarly, $C^*(\xi_x + f_x,\xi_y + f_y)$ is –1 order term situated at $(-f_x, -f_y)$ which carry complex conjugate information about of the specimen. After applying Fourier filtering of zero and – 1 order terms, Eq. 4 can be reduced into the following form:

$$F(\xi_x,\xi_y) = C(\xi_x - f_x,\xi_y - f_y) \quad (5)$$

The filtered spectrum is, first, shifted at the origin and then inverse Fourier transformed to retrieve the complex signal $f(x,y)$,

$$f(x,y) = c(x,y) = b(x,y)\exp(i\phi(x,y)) \quad (6)$$

subsequently the wrapped phase map from the following expression[2, 3]:

$$\phi(x,y) = \tan^{-1}\left[\frac{Im(f(x,y))}{Re(f(x,y))}\right] \quad (7)$$

where Im and Re are the imaginary and real part of the complex signal. The reconstructed wrapped phase map lies between $-\pi$ to $+\pi$. These discontinuities are then corrected by minimum L^P–norm two dimensional (2D) phase unwrapping algorithm[4]. The retrieved unwrapped phase map can be further used to calculate corresponding height map 'h(x, y)' by using the following expression[3]:

$$h(x,y) = \frac{\lambda}{4\pi\Delta n}\phi(x,y) \quad (8)$$

where, Δn is the difference between refractive indices of cell and outside medium. λ is the peak wavelength of the light source (λ = 632 nm for He-Ne laser).

Waveguide trapping theory of RBC deformation:

The phenomenon of RBC deformation can be explained using the finite element numerical simulations of optical forces and optical pressure of RBC trapped on top of waveguide surface[5]. The optical force can be found by integrating the Maxwell stress tensor T over the cell surface S [6, 7]:

$$\vec{F} = \oint_S \overleftrightarrow{T} \cdot \hat{n}\, dS. \quad (9)$$

where the outwardly directed unit normal on the surface is given by $\vec{n}$. It has been shown previously that the determination of optical forces using local optical pressure instead of using Maxwell stress tensor approach results in smaller noise and use smaller computational time[5, 8]. The force density f on a non-magnetic, isotropic dielectric body in an electromagnetic field is [9, 10]:

$$f = -\frac{1}{2}\epsilon_0 E^2 \nabla\epsilon \quad (10)$$

with ϵ_0 denoting the electric permittivity of vacuum, E the electric field and $\nabla\epsilon$ is the permittivity gradient.

It was reported that the net effect of the force density (i.e. pressure multiplied with surface normal) was to straighten the part of the cell that is overlapping with the evanescent field and make it parallel to the waveguide[5]. Although, the largest force component was to attract the RBC towards the surface. As the cell is an elastic body, the net effect of the optical forces (force density) is to press the cell downwards and to straighten the part of the cell that is interacting with the evanescent field and make it parallel to the waveguide. When this part of the cell is becoming straight, the rest of the cells deform and a change in the shape occurs. The elastic membrane of the RBC plays a crucial role in spreading the localized optical pressure over the entire cell[11].

Influence of the trapping beam (i.e. IR light) on the phase measurement

We systematically studied the influence of IR wavelength on phase measurement results. The trapping experiments of RBCs with and without IR filter both are performed, which is inserted into the beam path just before the camera. Any significant change in the recovered phase maps of trapped RBC with and without IR filter is not observed. However, it is observed that the use of IR filter degrades the interferogram quality and subsequently reduces the spatial phase sensitivity of the system due to multiple reflections of laser light between top and bottom surfaces of IR filter. Therefore, insertion of IR filter into the beam path is not preferred.

The IR beam does not influence the phase measurement results because of the following reasons: (1) there is no coherence between IR (used for trapping), and red (used for phase imaging) because both the light sources emit statistically independent radiation. Therefore, IR beam would not interfere with He-Ne laser beam and subsequently does not influence the phase results, (2) in our proposed system; IR light beam is not directly reaching at the camera plane. The IR photons scattered due to the roughness of waveguide and specimens (RBC) and un-scattered photons are actually reaching at the camera plane, which are very less in numbers. To see these photons at the camera plane, the camera is run at maximum exposure and gain. However, the numbers of photons due to phase imaging laser (He-Ne laser @ 632 nm wavelength) are quite high, which increases the signal to noise (SNR) of interferogram and subsequently reduces the influence of IR light on the phase measurements of RBCs. In case of IR leaking to the camera, it will increase the DC level in the interferogram and results a decrease in the reconstructed phase sensitivity and (3) spectral response of the CMOS camera for IR wavelength (~1070 nm) is quite poor (quantum efficiency (QE) < 5%). However, QE of the camera is maximum at 600 nm wavelength. This again increases the SNR of recorded interferogram at 632 nm and reduces the effect of IR wavelength.

If other trapping wavelength (say 532nm) is used for all trapping experiments, then a narrow bandpass green filter would be needed into the beam path as the QE of the camera at 532 nm wavelength is quite high (~ 70 %). However, this may reduce the spatial phase sensitivity of QPM due to multiple reflections inside the green filter.

Spatial phase sensitivity of QPM:

The smallest morphological changes that can be measured by the proposed technique are defined by the phase/height sensitivity of the system. The spatial phase/height measurement sensitivity of the system is quantified using standard flat mirror and waveguide substrate as a test specimen.

First, spatial phase sensitivity of the system is measured with a standard optical flat mirror (Size: 1"; Flatness: λ/10 @ 633 nm; Supplier: Thorlabs) placed under proposed QPM. Supplementary Fig. S1a illustrates the interferogram of flat mirror. The recorded interferogram is processed using Fourier Transform fringe analysis algorithm for the retrieval of phase and subsequently height map of flat mirror. The phase and height map of the standard flat mirror are depicted in Supplementary Figs. S1b and S1c. The spatial phase and height measurement sensitivity of the system thus obtained is found to be equal to 24.2 mrad and 1.2 nm. Next, experiment is performed with waveguide chip (see Fig.1c in the main text) for the measurement of spatial phase sensitivity and compared with the results obtained for flat mirror. Supplementary Figs. S1d – S1f depict the recorded interferogram, recovered phase and subsequently measured height variation of the waveguide chip. The spatial phase and height measurement sensitivity of the system in case of waveguide is found to be equal to 50.9 mrad and 2.6 nm respectively.

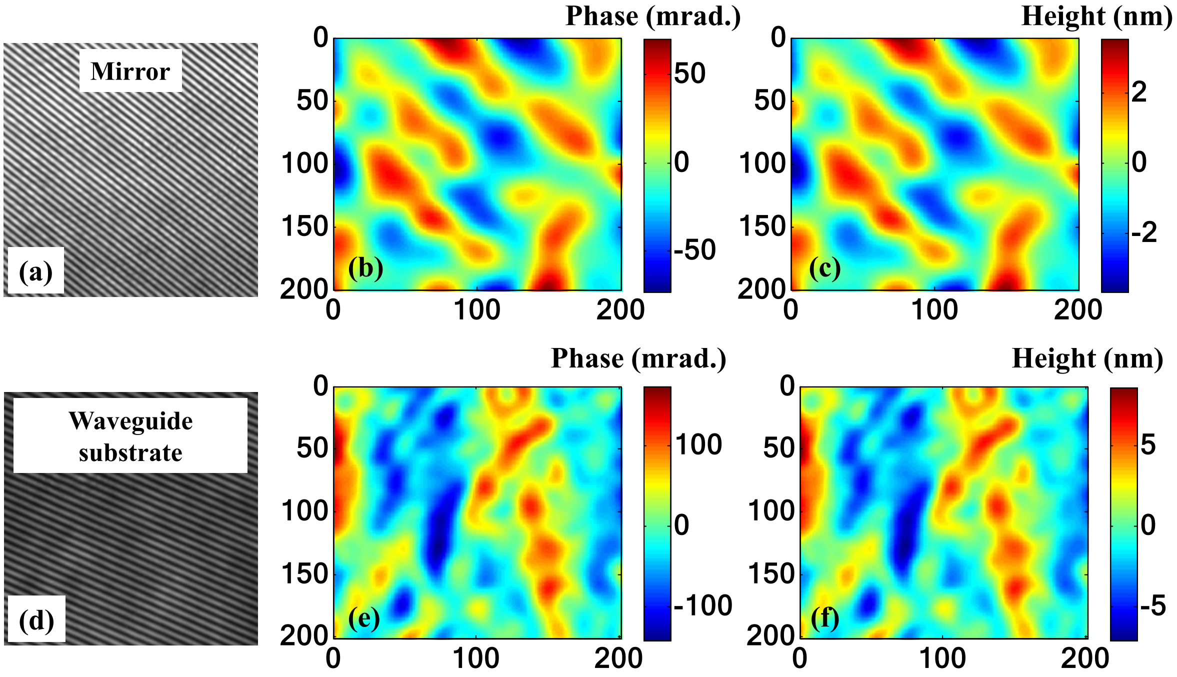

Supplementary Fig. S1. Comparison of spatial phase sensitivity of the system in case of flat mirror and waveguide as a test specimen. (a, d) Interferograms, (b, e) recovered phase maps and (c, f) corresponding height maps obtained for flat mirror and waveguide substrate, respectively.

Table S1 shows the comparison of spatial phase and height measurement sensitivity while using flat mirror and waveguide as test specimens. The peak to valley (P-V) error in the phase/height measurement for waveguide is observed to be approximately twice than that for standard mirror. This could be due to the multiple reflections from the ~2 μm thick SiO_2 layer over the Si substrate (see Fig.1c in the main text).

Table S1. Variation of Standard deviation (SD) and peak to valley (P-V) error of the spatial phase/height measurement sensitivity of the system in case of flat mirror and waveguide as a test specimen.

**Test object**	**Phase sensitivity (mrad.)**		**Height measurement sensitivity (nm)**	
	SD	P-V error	SD	P-V error
Mirror	24.2	143.1	1.2	7.2

Waveguide	50.9	313.3	2.56	15.8

The P-V spatial phase sensitivity of the system during optical trapping is found to be equal to ~ 313 mrad, which corresponds to the height measurement accuracy of 16 – 20 nm. The spatial phase sensitivity is observed to be less during optical trapping than that for standard flat mirror. This could be due to the formation of unwanted spurious fringes, which arise due to multiple reflections from several layers of waveguide structures, water-coverslip and air-coverslip interfaces. This is further confirmed experimentally by employing the system for the height measurement of 20 nm rib height waveguide. The 20nm rib height of optical waveguide is not visible, i.e., embedded with phase noise of the system, in the reconstructed RBCs phase map (Supplementary Fig. S2a). However, a 50nm rib height introduces a constant waveguide phase into the RBC's phase maps as depicted in Supplementary Fig. S2b.

To measure the height measurement accuracy of the system during trapping, experiment is conducted on a 50 nm rib height waveguide. Supplementary Figs. S3a – S3c present the interferogram, recovered phase and subsequently measured height map of the 50 nm optical waveguide. The height of the waveguide is measured to be equal to 49.7 $\pm$ 2.5 nm by employing the present system. Thus, the height measurement accuracy of the system lies between 20 and 50 nm. Therefore, the proposed technique has the capability to measure small morphological change (> 20 nm) in the height of RBCs or any other cell.

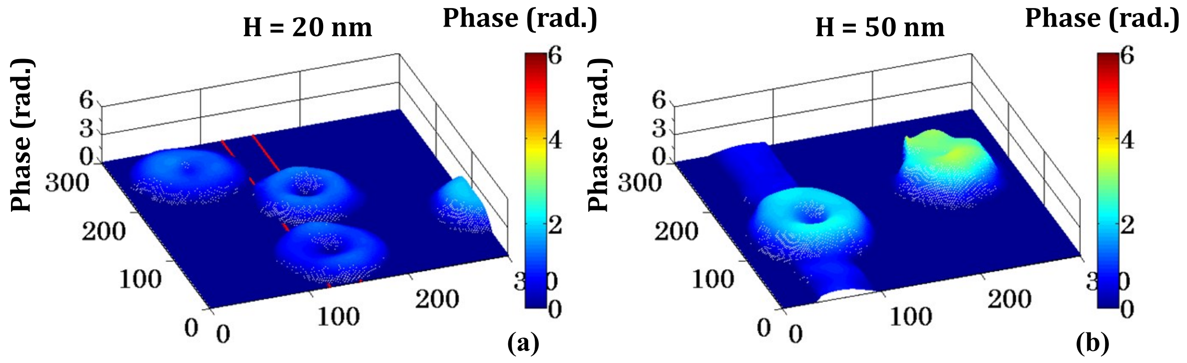

Supplementary Fig. S2. Quantitative phase imaging of human RBCs when sample is prepared on an optical waveguides having rib height of 20 nm and 50 nm. (a) Recovered phase map of RBCs without the appearance of 20 nm waveguide. (b) Recovered phase map of RBCs with 50 nm rib height waveguide in the background.

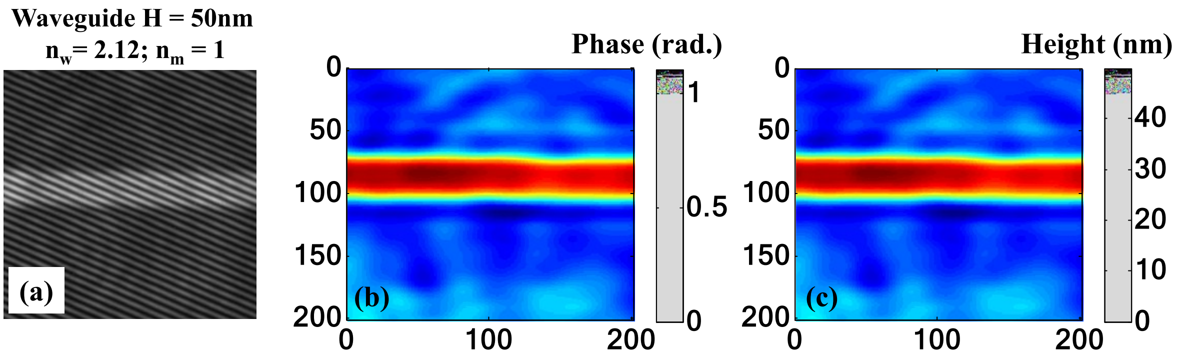

Supplementary Fig. S3. Measurement of phase/height measurement accuracy of the system using 50 nm thick rib waveguide as a test specimen. (a) Interferogram, (b) recovered phase map and (c) corresponding height map obtained for 50 nm thick standard rib waveguide.

Comparison of strip and rib waveguides:

The propagation losses as a function of waveguide width for the strip and rib waveguide structures have been extensively studied[12, 13]. It has been reported that the propagation losses are higher for narrow strip waveguide than for rib waveguide structures due to the roughness of the etched sidewalls[13, 14]. In the present study, a comparative analysis of the strip and rib waveguide geometries was performed to obtain the best fit for the waveguide trapping and QPM integrated setup.

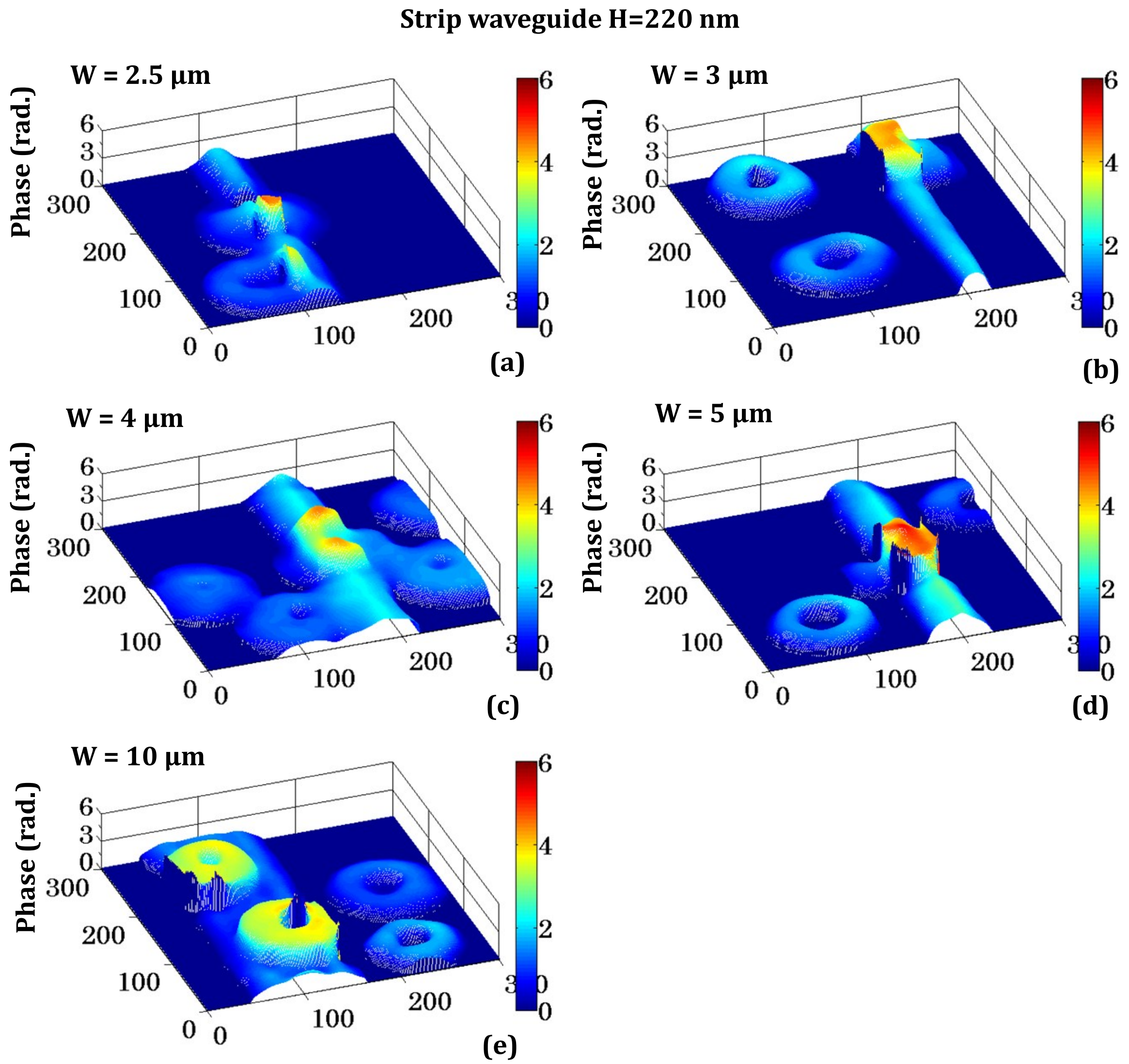

Supplementary Fig. S4. Phase maps of the RBCs with the waveguide background information of strip waveguides having widths:(a) 2.5, (b) 3, (c) 4, (d) 5, (e) 10 μm and height H=220 nm.

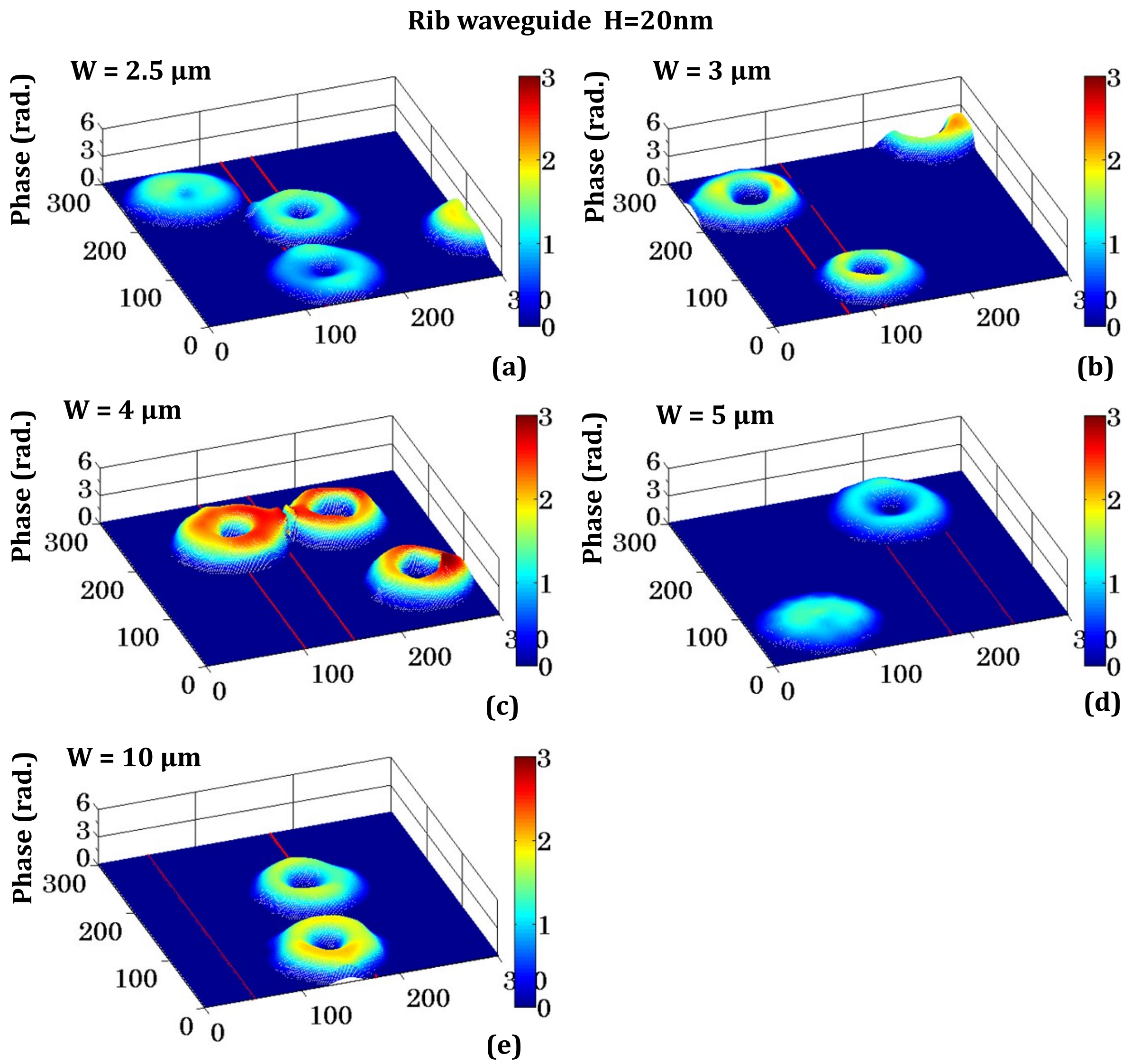

Supplementary Fig. S5. Phase maps of the RBCs with the rib waveguide structure having height H = 220 nm and varying waveguide widths: (a) 2.5, (b) 3, (c) 4, (d) 5, and (e) 10 μm.The straight red lines were used to depict the position of rib waveguides.

First, a comparative study of the strip (H = 220 nm) and rib (H = 20 nm) waveguide having different widths (w = 2.5, 3, 4, 5, 10 μm) was made. Supplementary Figs. S4 and S5 show the total phase value, i.e., RBC + waveguide phase, for two different waveguide geometries. It is evident from the phase images shown in Supplementary Fig. S4 (a – e) that for the strip waveguides (H=220 nm), phase reconstruction of the RBCs are noisy at the edges of the waveguide, this was due to its abrupt change in the phase values at the edges. The edges of the waveguide suffers from sidewall roughness due to the fabrication process (ion-beam etching)[15]. Moreover, the sidewall roughness is random in nature along the length of the waveguide. Strip waveguide has thickness of 220nm, while the rib height is only 20 nm for rib waveguides. Consequently, there is far less sidewall roughness and smaller abrupt phase change at the edges for a rib waveguide as compared to a strip waveguide. Therefore, rib waveguides do not suffer from all the phase errors at the edges of the waveguide, thus producing a smooth RBC phase map as shown in Supplementary Fig. S5 (a –

e).Rib waveguides were therefore preferred over strip waveguide for this work. Two straight red lines in the phase images were drawn to show the position of the rib waveguide, which otherwise is not visible due to small rib height of 20 nm.

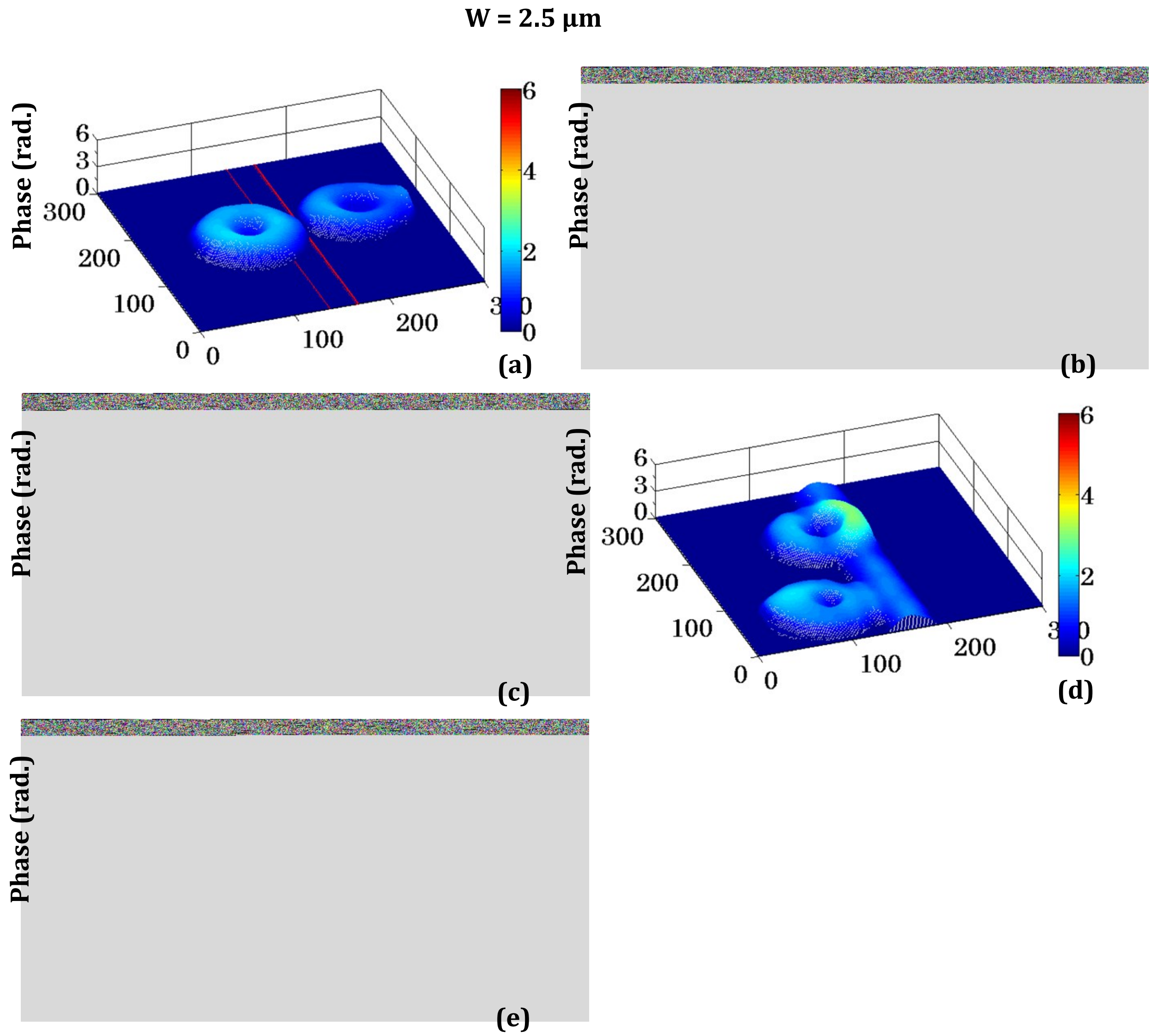

Supplementary Fig. S6. Reconstructed phase maps of the RBCs for different waveguide heights; (a) 8, (b) 20, (c) 50, (d) 150, and (e) 220 nm, having 2.5 µm waveguide width. The straight red lines in few phase images depict the position of the waveguide.

Next, the effect of waveguide height on the phase images was studied for a given width of the waveguide (of 2.5 µm). Supplementary Fig. S6 (a – e) shows the phase images of RBCs on waveguides of different heights (H = 8, 20, 50, 150, 220 nm). It was depicted from Supplementary Fig. S6 (a) and (b) that there was negligible influence on the RBC phase images by the waveguide with rib height of 8 – 20 nm, while the phase artifacts started to appear when waveguides with rib height above 50 nm were used. Consequently, for all the studies in this work rib waveguide with rib height of 20 nm was chosen.

Defocus correction algorithm:

Before the measurement of various RBCs morphological parameters, a focus correction algorithm is employed for the accurate phase measurement. Focus correction algorithm is implemented by taking the Fourier transform of the complex signal '$f(x,y;0)$' to obtain an angular spectrum[16, 17].

$$A(f_x,f_y;0) = \iint_{-\infty}^{\infty} f(x,y;0)e^{-j2\pi(f_x x + f_y y)}dxdy \qquad (11)$$

where, $f_x = \alpha/\lambda$ and $f_y = \beta/\lambda$ are the transform variables in terms of direction cosines α, β of the wave vector with respect to x, y axis and wavelength λ. The angular spectrum $A(f_x,f_y;0)$ is then propagated along the propagation direction 'z-axis' to obtain the field at different plane situated at a distance 'z' by using the following expression[17]:

$$A(f_x,f_y;z) = A(f_x,f_y;0)T(f_x,f_y) \qquad (12)$$

where, $T(f_x,f_y)$ is the freespace transfer function which is defined as follows[17]:

$$T(f_x,f_y) = \begin{cases} e^{-j\frac{2\pi z}{\lambda}\sqrt{1-(\lambda f_x)^2-\lambda f_y{}^2}}, & \sqrt{f_x^2+f_y^2} < \frac{1}{\lambda} \\ 0, & otherwise \end{cases} \qquad (13)$$

Then, inverse Fourier transform is done to obtain complex field '$f(x,y;z)$' in spatial domain at z distance:

$$f(x,y;z) = \iint_{-\infty}^{\infty} A(f_x,f_y;z)e^{j2\pi(f_x x + f_y y)}df_x df_y \qquad (14)$$

$f(x,y;z)$ is the calculated complex field at a distance z. This can be further used to calculate the accurate phase/height maps related to specimens during their holographic phase imaging if the accurate defocus distance is known.

To calculate the defocus distance the complex field '$f(x,y;0)$' obtained from the Fourier transform method (Eq. 6) is propagated across a range of – d μm to + d μm by following the steps mentioned above. The amplitude variance 'M_{var}^{d}' is then calculated corresponding to each propagated complex field $f(x,y;d)$ by using the following expression[17]:

$$M_{var}^{d} = \frac{1}{\mu}\sum\sum[|f(x,y;d)| - \mu]^2 \qquad (15)$$

where μ is the mean of the amplitude of the complex field at distance d. The sharpness curve is formed by plotting the amplitude variance as a function of reconstruction distance. The minimum of the sharpness curve gives information about the defocus distance or true focal plane of the objective lens. Finally, the complex field '$f(x,y;0)$' is propagated by above calculated defocus distance using the steps given above to accurately calculate the phase/height maps of the specimens.

Influence of defocus on the RBCs phase images:

Supplementary Fig. S7 clearly depicts the influence of defocus on the extracted RBC phase map. The recorded interferogram correspond to human RBCs is shown in Supplementary Fig. S7a. As can be seen, if we employ Fourier transform based phase recovery algorithm without focus correction, then defocused RBC phase information is reconstructed as illustrated in Supplementary Fig. S7b, which greatly influence the measurements related to RBC's morphological parameters.

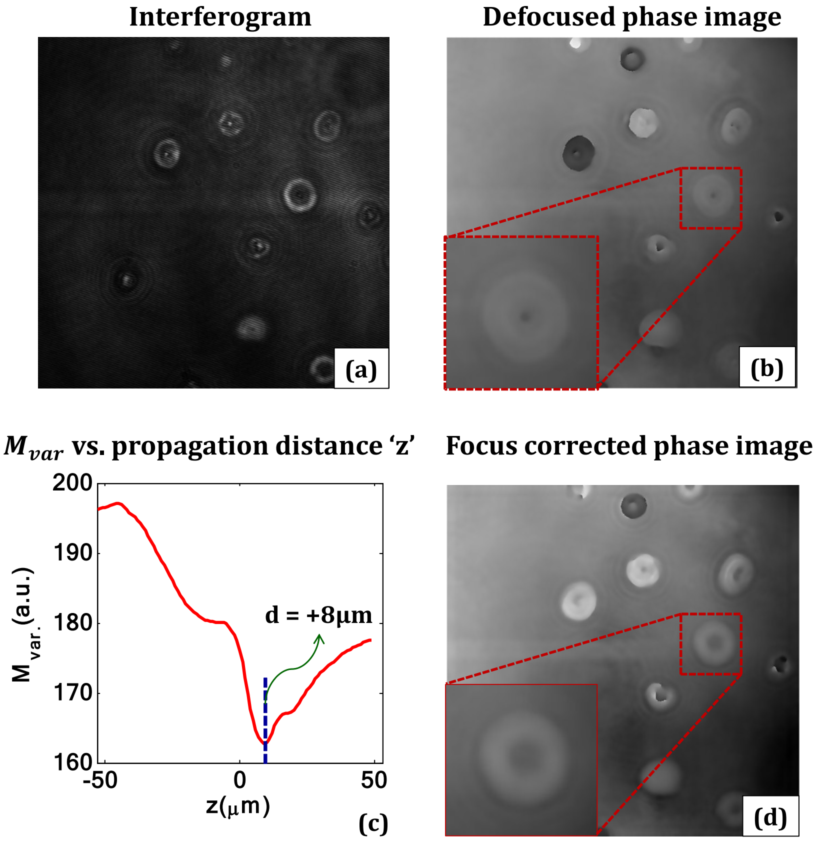

Supplementary Fig. S7. Defocus correction of the human RBC's reconstructed phase images. (a) Interferogram of human RBCs. (b) Reconstructed phase maps of human RBCs without focus correction. (c) Sharpness curve of amplitude variance as a function propagation distance z. (d) Reconstructed phase maps of human RBCs with focus correction using the angular spectrum propagation approach.

Therefore, it is mandatory to accurately quantify amount of defocus present in recorded interferograms. Supplementary Fig. S7c is the sharpness curve depicting the variation of amplitude variance 'M_{var}^{d}' (calculated using Eq. 15) as a function of propagation distance 'z'. It can be envisaged from Supplementary Fig. S7c that minimum of sharpness curve lies at z = + 8 μm, which gives information about true focal plane of the objective lens. The defocused RBC phase information is further refocused by propagating the complex field from the reconstruction plane to the focal plane of the objective lens (i.e., d = +8 μm) using angular spectrum propagation approach. After digital refocusing of the complex field, nice RBC phase map having perfect donut shape is reconstructed (Supplementary Fig. S7d).

Additional experimental result:

During planar trapping and transportation of the RBC, the size of central donut region was decreased, while the size of the cell was slightly increased. Supplimentry Fig. S8 shows the phase images of the untrapped and trapped RBC from another series of experiments that provide a better overview about the change in maximum phase value in 3D view and the increase in the size of the RBC. The upper two rows of Supplementary Fig. S8 depict the quantitative phase images of the untrapped RBC (laser is off), whereas, the lower two rows represent the phase images of the trapped RBC, while propelling it on the top surface of the waveguide. In Supplimentry Fig. S8 the phase images of the RBC are obtained from the interferometric movie by selecting the region of interest and obtaining the phase at different time points. Prior to switching on the laser at t= 17 sec, there was little variation in the phase valuse of the RBC. It can be noticed that during trapping, the maximum phase value of RBC decreases.

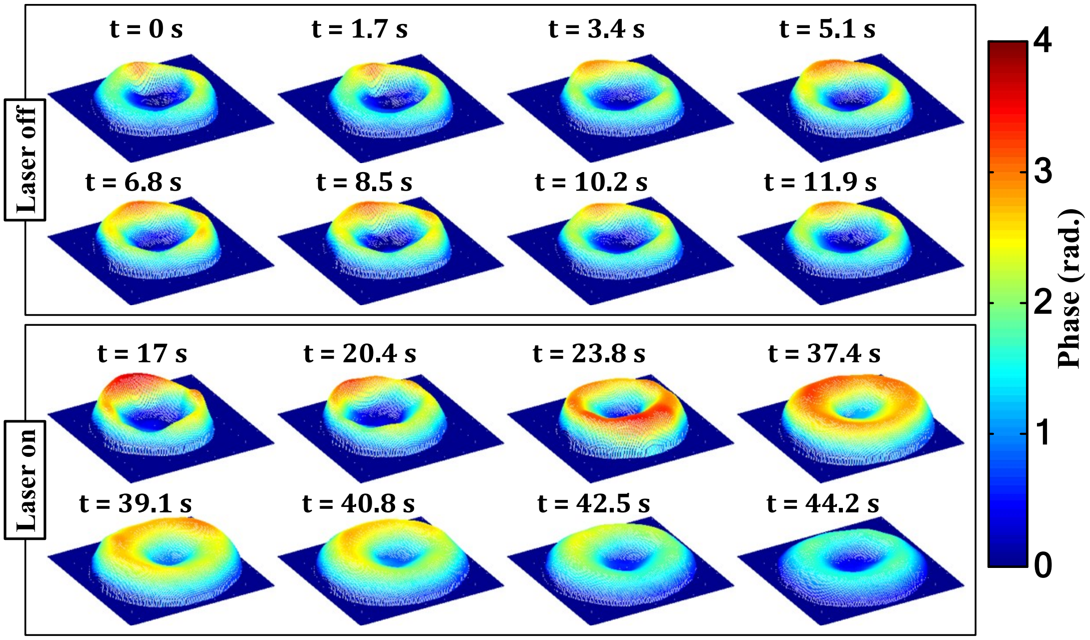

Supplementary Fig. S8. Time lapsed quantitative phase images of untrapped (upper two rows) and trapped (Lower two rows) RBC. The color bar is in radians.

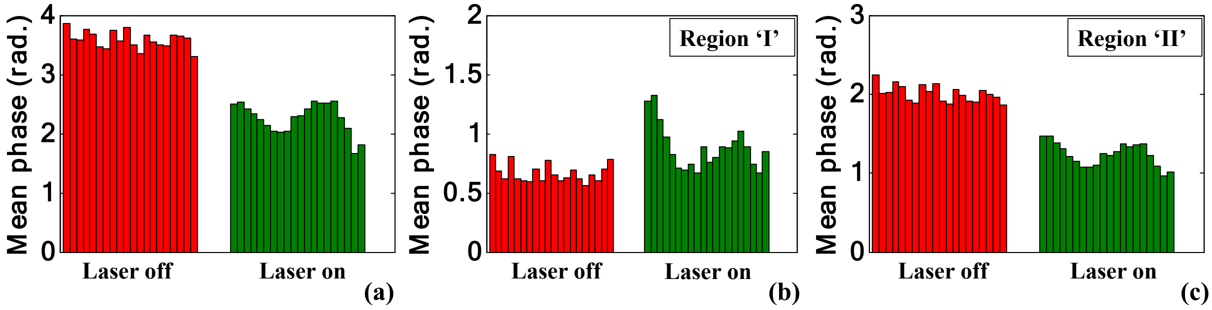

Supplementary Fig. S9. Bar plots (a) of the maximum phase values (φ_{max}) and (b, c) of the mean phase values of RBC's time lapsed frames of region I and region II (shown in Fig. 4a) for laser off (0 – 14s frames) and on (26 – 40s frames) conditions for a single movie (experiment).

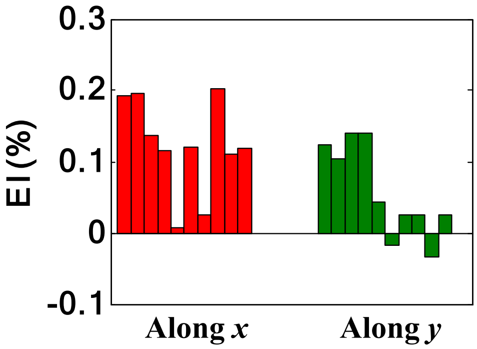

Supplementary Fig. S10. The bar plot of human RBC's EI along x and y axis during waveguide trapping for 9 different experimental series.

Quantification of cell's surface area:

The total surface area 'S' of the RBC's membrane is the sum of all the area elements 'A_1' and projected area 'A_2' as illustrated in Supplementary Fig. S11, which is calculated by utilizing Eq. 20.

The area element of the cell's top surface

$$dA_1 = dx_1 dy_1 \tag{16}$$

The sum of all the area elements

$$A_1 = \sum_i dA_i = \sum_i dx_i dy_i \tag{17}$$

The projected area element of the cell's top surface on $x - y$ plane

$$dA_2 = dx_2 dy_2 \tag{18}$$

The sum of all the projected area elements

$$A_2 = \sum_j dA_j = \sum_j dx_j dy_j \tag{19}$$

The total surface area 'S' of RBC's membrane:

$$S = A_1 + A_2 \tag{20}$$

Thus, 3D cell's information is utilized for all the calculations related to morphological parameters such as surface area, volume, surface area to volume ratio and sphericity.

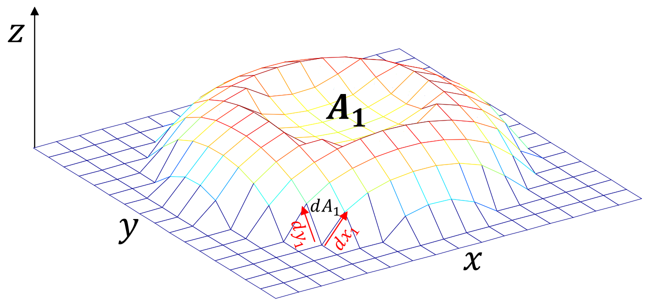

Supplementary Fig. S11. Quantification of cell's membrane surface area.

References:

1. Z. Yaqoob, T. Yamauchi, W. Choi, D. Fu, R. R. Dasari and M. S. Feld, *Optics Express*, 2011, **19**, 7587-7595.
2. M. Takeda, H. Ina and S. Kobayashi, *JosA*, 1982, **72**, 156-160.
3. G. Popescu, *Quantitative phase imaging of cells and tissues*, McGraw Hill Professional, 2011.
4. D. C. Ghiglia and L. A. Romero, *J. Opt. Soc. Am. A*, 1996, **13**, 1999-2013.
5. B. S. Ahluwalia, P. McCourt, A. Oteiza, J. S. Wilkinson, T. R. Huser and O. G. Hellesø, *Analyst*, 2015, **140**, 223-229.
6. S. Gaugiran, S. Gétin, J.-M. Fedeli, G. Colas, A. Fuchs, F. Chatelain and J. Dérouard, *Opt. Express*, 2005, **13**, 6956-6963.
7. J. D. Jackson, *Classical electrodynamics*, Wiley, 1999.
8. O. G. Hellesø, *Applied optics*, 2017, **56**, 3354-3358.
9. I. Brevik, *Physics Reports*, 1979, **52**, 133-201.
10. I. Brevik and R. Kluge, *JOSA B*, 1999, **16**, 976-985.
11. M. Dao, C. T. Lim and S. Suresh, *J. Mech. Phys. Solids*, 2003, **51**, 2259-2280.
12. L. Vivien, F. Grillot, E. Cassan, D. Pascal, S. Lardenois, A. Lupu, S. Laval, M. Heitzmann and J.-M. Fédéli, *Optical Materials*, 2005, **27**, 756-762.
13. S. M. Lindecrantz and O. G. Hellesø, *IEEE Photonics Technology Letters*, 2014, **26**, 1836-1839.
14. B. S. Ahluwalia, Ø. I. Helle and O. G. Hellesø, *Optics Express*, 2016, **24**, 4477-4487.
15. B. S. Ahluwalia, A. Z. Subramanian, O. G. Hellso, N. M. Perney, N. P. Sessions and J. S. Wilkinson, *IEEE Photonics Technology Letters*, 2009, **21**, 1408-1410.
16. M. T. Rinehart, H. S. Park and A. Wax, *Biomed. Opt. Express*, 2015, **6**, 2067-2075.
17. M. Doğar, H. A. İlhan and M. Özcan, *Review of Scientific Instruments*, 2013, **84**, 083704.
